# eEF2K-Mediated Stabilization of PCBP2 Promotes Oncogenic mRNA Programs in Triple-Negative Breast Cancer

**DOI:** 10.7150/ijbs.127111

**Published:** 2026-03-25

**Authors:** Yueying Cheng, Ting Jiang, Wenqian Zhu, Linhao He, Zongling Chen, Hang Li, Ruigang Zhao, Shilong Jiang, Yan Cheng

**Affiliations:** 1 Department of Pharmacy, The Second Xiangya Hospital, Central South University, Changsha, 410011, China.; 2 Hunan Provincial Engineering Research Centre of Translational Medicine and Innovative Drug, Changsha 410011, China.; 3 Department of Medical Administration, The Second Hospital of Shandong University, Jinan, 250033, China.; 4 Department of General Surgery, The Second Xiangya Hospital, Central South University, Changsha, Hunan, 410011, China.; 5 Information and Network Center, Central South University, Changsha 410083, China.; 6 Department of Pharmacy, Xiangya Hospital, Central South University, Changsha, 410008, China.; 7 The Hunan Institute of Pharmacy Practice and Clinical Research, Xiangya Hospital, Central South University, Changsha, Hunan, 410008, China.; 8 Clinical Research Center for Breast Disease in Hunan Province, Changsha, 410011, China.; 9 FuRong Laboratory, Changsha 410078, Hunan, China.; 10 NHC Key Laboratory of Cancer Proteomics & State Local Joint Engineering Laboratory for Anticancer Drugs, Xiangya Hospital, Central South University, Changsha 410008, Hunan, China.; 11 Key Laboratory of Diabetes Immunology (Central South University), Ministry of Education Changsha, 410011, China.

**Keywords:** triple-negative breast cancer, eEF2K, PCBP2, mRNA stability, ubiquitin-proteasome degradation

## Abstract

Triple-negative breast cancer (TNBC), characterized by the absence of effective therapeutic targets, remains a major clinical challenge with poor prognosis. The identification of novel molecular targets is therefore crucial for developing effective treatment strategies. Eukaryotic elongation factor 2 kinase (eEF2K) is highly expressed in TNBC and known to promote tumor progression; however, the precise mechanisms underlying its oncogenic role remain elusive. In this study, we identified poly(rC)-binding protein 2 (PCBP2) as a previously unrecognized downstream substrate of eEF2K. Analysis of clinical TNBC specimens revealed a positive correlation between eEF2K and PCBP2 protein expression levels. Further studies demonstrated that site-specific phosphorylation of PCBP2 at serine 189 (Ser189) markedly promoted the malignant phenotype of TNBC cells. Mechanistically, eEF2K-mediated phosphorylation at Ser189 stabilized PCBP2 by preventing its ubiquitin-proteasome-dependent degradation. This phosphorylation-dependent stabilization, in turn, enabled PCBP2 to promote the mRNA stability of pro-oncogenic genes, including TNC, SOX5, and ITGB3, thereby driving TNBC progression. Collectively, these findings not only reveal PCBP2 as a critical downstream effector of eEF2K, but also highlight the eEF2K-PCBP2 signaling axis as a promising therapeutic target for TNBC.

## Introduction

Globally, breast cancer continues to be the most commonly diagnosed malignant disease and the primary cause of cancer-related deaths in women 1. Among its subtypes, triple-negative breast cancer (TNBC)—characterized by lack of expression of estrogen receptor (ER), progesterone receptor (PR), and human epidermal growth factor receptor 2 (HER2)—stands out as the most aggressive form, linked to the worst clinical prognosis 2, 3. This subtype exhibits distinct features: notably shorter survival durations, highly invasive tumor behavior, and a pronounced tendency for distant metastasis compared to other breast cancer types 4. At present, neoadjuvant chemotherapy combined with surgical resection remains the mainstay of treatment. However, approximately 30% of patients relapse after surgery, nearly half eventually develop chemoresistance 5-7. Consequently, there is a critical imperative to uncover the molecular mechanisms driving TNBC pathogenesis, thereby enabling the development of innovative therapeutic approaches.

Eukaryotic Elongation Factor 2 Kinase (eEF2K), a calcium/calmodulin- dependent α-kinase family member, phosphorylates eEF2 at Thr56 under nutrient-limiting conditions, thereby suppressing translation elongation 8, 9. Emerging evidence demonstrates that eEF2K functions as an oncogene, exhibiting significant overexpression across multiple malignancies, including breast cancer, pancreatic carcinoma, glioblastoma and lung carcinoma. Clinically, heightened eEF2K expression correlates with reduced overall survival in patient cohorts 10-14. Our previous work demonstrated that eEF2K promotes proliferation of breast cancer cells through reprogramming metabolism toward aerobic glycolysis 15, and that targeting eEF2K can sensitize breast cancer cells to conventional chemotherapy 16. While the upstream regulatory mechanisms of eEF2K have been relatively well-characterized, with mTORC1 17 and p90RSK 18 directly phosphorylating eEF2K at Ser78 and Ser366 residues, respectively, the downstream signaling networks remain largely underexplored. To date, eEF2 represents the most well-documented phosphorylation substrate of eEF2K, playing a critical role in regulating ribosomal translocation during translational elongation 19. Our recent studies have unveiled additional downstream targets, demonstrating that: 1) eEF2K-mediated inhibition of GSK3β at Ser9 elevates PD-L1 expression, fostering tumor immune escape 20; 2) eEF2K phosphorylates AURKA at Ser391, thereby stabilizing AURKA protein levels and promoting the malignant progression of TNBC 21, indicating eEF2K may be involved in various biological behaviors through wide substrates. Therefore, systematic exploration of downstream effector network of eEF2K is essential for elucidating its multifaceted oncogenic functions and identifying novel therapeutic targets.

Poly(rC)-binding protein 2 (PCBP2), an RNA-binding protein with high sequence-specific affinity for poly(C) motifs 22, engages in diverse regulatory processes such as pre-mRNA splicing, mRNA stabilization, and translational control 23, 24. Initially, its role in mRNA stability was identified through studies on α-globin mRNA, where the KH domains of PCBP2 bind tightly to the pyrimidine-rich 3' untranslated region (3'UTR), thereby increasing transcript stability 25. A similar mechanism operates for neurofilament mRNA, where PCBP2 interaction with its 3'UTR also promotes stabilization 26. Emerging evidence indicates that PCBP2 plays an oncogenic role across various human cancers, where its overexpression drives tumor progression and metastatic spread 27. For example, in hepatocellular carcinoma, elevated PCBP2 expression contributes to the development of sorafenib resistance 28, while in glioblastoma, higher PCBP2 levels correlate with advanced tumor grade and significantly poorer prognosis 29. Likewise, breast carcinoma tissues show markedly elevated PCBP2 expression relative to adjacent normal tissues, linking it to disease advancement and unfavorable clinical outcomes 30. These collective findings posit PCBP2 as a potential therapeutic target in multiple malignancies. It has been reported that PCBP2 undergoes predominantly phosphorylation as its major post-translational modification 31. However, the specific kinases responsible for PCBP2 phosphorylation and the mechanisms underlying its subsequent regulatory effects on PCBP2 function remain to be systematically investigated.

Our study identifies PCBP2 as a potential substrate of eEF2K and demonstrates that eEF2K-mediated phosphorylation at Ser189 protects PCBP2 from proteasomal degradation, thereby markedly enhancing its protein stability. RNA sequencing analysis further reveals that eEF2K and PCBP2 coordinately regulate downstream targets—such as tenascin-C (TNC), SRY-box transcription factor 5 (SOX5), and integrin β3 (ITGB3)—through modulation of mRNA stability. Collectively, these findings uncover a previously unrecognized regulatory axis in which eEF2K-dependent phosphorylation of PCBP2 stabilizes the protein and, in turn, governs the expression of critical genes driving tumor progression. This work not only broadens the spectrum of known eEF2K substrates but also provides mechanistic insights into the therapeutic potential of targeting eEF2K signaling in TNBC.

## Material and Methods

### Cell lines and culture

In this study, four human cell lines were employed: BT549 and HCC1806, both derived from breast cancer, were cultured in RPMI-1640 medium (Gibco), while HEK-293T and MDA-MB-231 cells were maintained in Dulbecco's Modified Eagle Medium (DMEM, Gibco). All culture media were supplemented with 10% fetal bovine serum (FBS) and antibiotics (100 units/mL penicillin and 100 μg/mL streptomycin). Cells were incubated at 37 °C in a humidified atmosphere containing 5% CO₂ and 95% air. To ensure authenticity, cell line identity was verified through short tandem repeat (STR) profiling, and experiments exclusively utilized cells within passages 3-20 post-thawing.

### Reagents and antibodies

The following reagents were obtained from commercial sources: cycloheximide (CHX) and MG132, protein synthesis inhibitor and proteasome inhibitor respectively, were acquired from Selleck Chemicals. Additionally, actinomycin D (HY-17559) was sourced from MedChemExpress (MCE). For immunoblotting analysis, primary antibodies were procured from multiple vendors: eEF2K (ab85721, 1:1000 dilution) was obtained from Abcam, PCBP2 (15070-1-AP, 1:1000) was purchased from Proteintech, GAPDH (GB11002, 1:5000) was acquired from Servicebio, and HA (3724s, 1:1000) and Flag (14793, 1:1000) antibodies were sourced from Cell Signaling Technology. Secondary detection reagents included peroxidase-conjugated AffiniPure Goat Anti-Rabbit IgG (H+L) and Anti-Mouse IgG (H+L), both purchased from Jackson Immuno Research.

### siRNA, shRNA, and plasmid transfections

Cells cultured in exponential growth phase were seeded at 1×10⁵ cells per well in six-well tissue culture plates and allowed to adhere for 24 hours before transfection. siRNA complexes were formed by combining siRNAs with Lipofectamine 2000 reagent and serum-free DMEM at a 1:1 ratio, following the manufacturer's detailed protocol. For stable PCBP2 knockdown, lentiviral particles containing PCBP2-targeted shRNA were used to transduce cells for 24 hours, followed by selection with 2 μg/mL puromycin to isolate stably transduced populations. Likewise, for stable expression of PCBP2-WT (wild-type) and PCBP2 Ser189Ala mutant (S189A), cells were infected with lentiviral vectors encoding the respective constructs. Transfection efficiency was evaluated through quantitative PCR or Western blot analysis, as recommended by the manufacturer's guidelines. The specific target sequences for siRNAs and shRNAs are listed in Table S1 (Supporting Information).

### RNA isolation, cDNA Synthesis, and real-time PCR analysis

RNA extraction was performed using TRIzol reagent (Invitrogen, USA). The isolated RNA was then converted into complementary DNA (cDNA) through reverse transcription with the PrimeScript RT Reagent Kit (Takara Bio). Real-time quantitative PCR (qPCR) analysis utilized SYBR Green dye (Takara Bio) on a QuantStudio Real-Time PCR System (Thermo Fisher Scientific, USA). Data collection and processing were carried out with QuantStudio Design & Analysis Software v1.5.1. Relative quantification of RNA levels was determined using the comparative threshold cycle (2^-ΔΔCt^) method.

### Western blot

Cellular proteins were extracted using RIPA buffer containing phosphatase and protease inhibitors, with lysis performed on ice. Following centrifugation at 12,000 rpm for 15 minutes at 4 °C, supernatants were collected for subsequent analysis. Protein samples (20-40 μg per lane) were denatured by boiling in Laemmli buffer and subsequently separated via SDS-PAGE. Proteins were then transferred to PVDF membranes. Membranes were blocked with 5% bovine serum albumin in TBST for one hour at room temperature. Primary antibodies were applied for overnight incubation at 4 °C. After washing steps, membranes were incubated with secondary antibodies for one hour at room temperature. Protein signals were detected using enhanced chemiluminescence reagent.

### Cell viability assays

Cell Counting Kit-8 (CCK-8, Bimake) was employed to evaluate cellular viability. Specifically, 3×10^3^ cells per well were plated in a 96-well plate (NEST Biotechnology, Wuhan, China) and maintained under standard culture conditions for 48 hours. Following this incubation period, 10 μL of CCK-8 solution was introduced into each well, and the cells were then incubated for an additional hour at 37°C. The optical density (OD) at 450 nm was subsequently measured using a microplate reader to quantify cell viability.

### Colony formation assay‌

To assess colony formation, cells were plated at 500 cells per well in 6-well tissue culture plates (NEST Biotechnology, Wuhan, China) and maintained in standard culture conditions for 15 days. After the incubation period, cells underwent fixation using 4% paraformaldehyde (Servicebio) for 15 minutes, followed by staining with 0.1% crystal violet for 20 minutes. Unbound dye was removed by thorough washing with phosphate-buffered saline (PBS). Subsequently, plates were air-dried, and images were captured for colony counting analysis.

### EdU incorporation assay

Cells were co-incubated with 50 µM 5-Ethynyl-2'-deoxyuridine (EdU, RiboBio) at 37°C for 2 hours following the manufacturer's instructions. Post-incubation, cells underwent fixation using 4% paraformaldehyde at room temperature for 30 minutes. Subsequent treatment involved incubation with 2 mg/ml glycine for 5 minutes followed by permeabilization with 0.5% Triton X-100 for 15 minutes at room temperature. Cells were then incubated with 1× Apollo reaction cocktail for 30 minutes and nuclei were counterstained with Hoechst 33342 (5 μg/mL) for 30 minutes at room temperature. Fluorescence images were captured using a confocal fluorescence microscope for analysis.

### Wound-healing assay

Cells were seeded in twelve-well plates and cultured until 90-100% confluence was achieved. A scratch wound was then created by mechanically scraping the cell monolayer with a sterile pipette tip. Cell migration was monitored at distinct time points (0 and 48 hours) using microscopic examination.

### Cell migration and invasion

Cells at a density of 2×10⁴ were seeded into the upper chamber of transwell inserts (8 μm pore size, Corning) within 24-well plates. The lower chamber was filled with either DMEM or RPMI-1640 medium containing 30% fetal bovine serum (FBS). For invasion experiments, the upper chamber was pre-coated with Matrigel (BD, Bioscience) before cell seeding. Plates were incubated at 37 °C with 5% CO₂ and 95% air for 48 hours (migration) or 72 hours (invasion). Following incubation, non-migrating or non-invasive cells in the upper chamber were eliminated using cotton swabs. Cells that migrated or invaded to the lower membrane surface were fixed with 4% paraformaldehyde and stained with crystal violet. Subsequently, migrated and invaded cells were visualized and quantified for analysis.

### RNA sequencing

Cell RNA sequencing was conducted with three biological replicates per experimental group by LC-Bio Technologies (LC-Bio Technologies, Hangzhou, China). Total RNA was extracted using TRIzol followed by ribosomal RNA depletion. The isolated RNA underwent fragmentation, end repair, adapter ligation, and PCR amplification according to the Illumina library construction protocol. Subsequently, the prepared libraries were sequenced on an Illumina HiSeq 3000 platform at the same facility.

### RNA pull-down assay

Target RNAs were initially synthesized using MAXIscript T7 In Vitro Transcript Kit (thermo AM1312) and subsequently 3'-end labeled with biotin through the Pierce RNA 3'End Desthiobiotinylation Kit (thermos 20163). The biotinylated RNAs were then incubated with lysates derived from MDA-MB-231 cells. RNA-protein complexes were isolated utilizing the Pierce Magnetic RNA-Protein Pull-Down Kit, adhering to the manufacturer's protocol (thermo 20164). Finally, specifically bound proteins were eluted by boiling the beads in 1× loading buffer for 10 minutes and subsequently analyzed via western blot.

### RIP assay

RNA immunoprecipitation (RIP) was carried out using the Magna RIP^TM^ RNA-Binding Protein Immunoprecipitation Kit (Millipore, Cambridge, MA), following the manufacturer's instructions. Briefly, cells were collected in IP lysis buffer and subjected to mechanical shearing for cell lysis. The resulting lysates were incubated overnight at 4 °C with either anti-PCBP2 antibodies or control IgG. Immune complexes formed were subsequently captured through incubation with streptavidin-coated magnetic beads for 2 hours. After this step, RNA was extracted and purified from the immunoprecipitates, followed by quantification via qRT-PCR analysis.

### Breast specimens, tissue microarray (TMA) and immunohistochemistry (IHC)

Clinical breast tissue specimens were obtained from the Second Xiangya Hospital of Central South University (Changsha, China). Prior to collection, written informed consent was obtained from all participating patients. To construct TMAs, paraffin-embedded samples were arranged using a dedicated arraying device, with each specimen represented by three replicate 1-mm-diameter cores to minimize tissue loss and address tumor heterogeneity. IHC staining was performed for PCBP2 (Proteintech, 15070-1-AP, 1:100 dilution) and eEF2K (Abcam, ab85721, 1:100 dilution) following the manufacturer's protocols with the DAKO LSAB + System-HRP kit (DAKO, Copenhagen, Denmark).

### Semi-quantitative evaluation of IHC staining

Two experienced board-certified pathologists, specializing in IHC, independently assessed all samples while remaining blinded to clinical patient information. The expression levels of eEF2K and PCBP2 were evaluated using a semi-quantitative scoring methodology. This approach integrated two key parameters: the proportion of immunoreactive cells and the staining intensity. For staining intensity, the scoring system assigned: 0 points for no detectable staining (negative), 1 point for light yellow (indicating weak positivity), 2 points for light brown (moderate positive), and 3 points for deep brown (strong positive). The percentage of positive cells was scored as follows: 1 point for ≤ 25% positive cells, 2 points for 26-50%, 3 points for 51-75%, and 4 points for > 75%. The final immunoreactivity score was derived by multiplying the intensity score by the percentage score, resulting in a range from 0 to 12. Based on this scoring, samples were classified into two groups: low expression (0-7) and high expression (8-12).

### Animal studies

All experimental protocols involving animals received approval from the Institutional Animal Care and Use Committee (IACUC) at Central South University (Approval ID: CSU-2024-0093). These procedures were carried out in full compliance with the institution's established guidelines for ethical animal treatment and research practices. For the tumor xenograft model, MDA-MB-231 cells (1×10^6^ per animal) stably expressing the designated constructs were prepared by suspending them in 100 µL of serum-free DMEM. Tumor progression was assessed through regular monitoring, including body weight measurements and caliper-based tumor volume calculations performed every two days. Tumor volume was determined using the formula (length×width²×π)/6, ensuring consistent evaluation. Following a two-week incubation period post-inoculation, tumors were surgically excised for subsequent histological and molecular analyses.

### Statistical analysis

Statistical analyses were performed utilizing GraphPad Prism 10.0. For pairwise comparisons between two groups, Student's t-test was employed, while multigroup analyses were conducted through one-way or two-way analysis of variance (ANOVA). All results are expressed as mean ± standard deviation derived from at least three independent experiments. A p-value below 0.05 was considered statistically significant.

## Results

### eEF2K interacts and positively correlates with PCBP2

**Figure 1:**
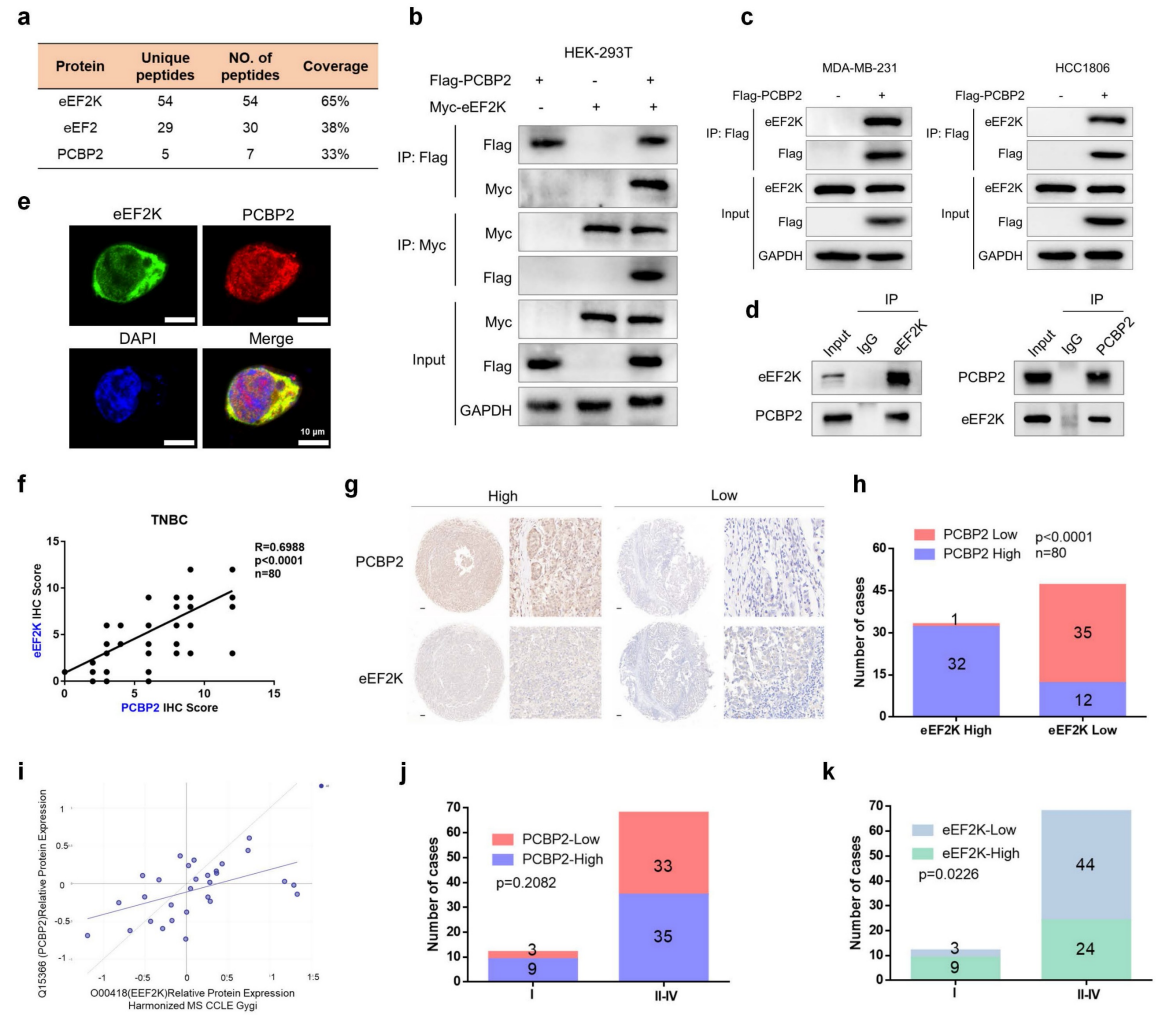
** eEF2K interacts and positively correlates with PCBP2. a**) The proteins immunoprecipitated by anti-Flag-eEF2K antibody were analyzed by mass spectrometry. **b**) HEK293T cells were transfected with Myc-eEF2K and Flag-PCBP2 plasmids, and then subjected to immunoprecipitation with anti-Flag or anti-Myc antibodies. The lysates and immunoprecipitates were then blotted. **c**) MDA-MB-231 and HCC1806 cells were transfected with Flag-PCBP2 plasmids, and then subjected to immunoprecipitation with anti-Flag antibodies. The lysates and immunoprecipitates were then blotted. **d**) MDA-MB-231 cell lysates were subjected to immunoprecipitation with IgG, anti-PCBP2 or anti-eEF2K antibodies. Immunoprecipitates were blotted with the indicated antibodies. **e**) The cellular location of eEF2K and PCBP2 in MDA-MB-231 cells was examined by immunofluorescence staining. DAPI was used to stain the nucleus. Scale bar, 10 μm. **f, g**) Correlation between IHC scores of eEF2K and PCBP2 from a tissue microarray containing 80 TNBC patients. Representative images of eEF2K and PCBP2 with low or high IHC scores. Semi quantitative scoring method (using a scale from 0 to 12) was used to analyzed the scores of PCBP2 and eEF2K IHC staining. Data analysis was performed using Pearson's correlation analysis. Scale bar, 100 μm. **h**) The number of cases of high or low PCBP2 in eEF2K high or low groups in this microarray. Data analysis was conducted through chi-square testing. **i**) The correlation between eEF2K and PCBP2 predicted by DepMap (r=0.505). **j, k**) The number of cases with high or low eEF2K and PCBP2 expression in stages I to IV. Data analysis was conducted through chi-square testing.

To further investigate eEF2K-related functional interactions, we performed IP followed by mass spectrometry to identify eEF2K-interacting proteins and identified PCBP2 to be a novel interacting partner of eEF2K (**Figure 1a**). This interaction was validated by reciprocal co-immunoprecipitation (co-IP) assays in HEK-293T cells co-transfected with Flag-PCBP2 and Myc-eEF2K, followed by western blotting, which confirmed their association (**Figure 1b**). Consistently, ectopic expression of Flag-PCBP2 in MDA-MB-231 and HCC1806 cells further demonstrated the specific interaction between PCBP2 and eEF2K (**Figure 1c**). Importantly, the endogenous interaction of the two proteins was also observed in MDA-MB-231 cells (**Figure 1d**). Complementary immunofluorescence microscopy revealed substantial co-localization of eEF2K and PCBP2 within the cytoplasm (**Figure 1e**). Collectively, these results establish PCBP2 as a bona fide interaction partner of eEF2K.

We next aimed to explore whether eEF2K and PCBP2 exhibit a functional correlation, IHC staining for eEF2K and PCBP2 were performed using TMA of 80 TNBC specimens. Staining intensity was scored according to established criteria, revealing a positive correlation between eEF2K and PCBP2 expression levels (**Figure 1f**). Immediately afterward, we divided eEF2K and PCBP2 into high and low expression groups based on scores (**Figure 1g**). Notably, within the eEF2K high-expression group, the proportion of cases with high PCBP2 expression significantly exceeded those with low PCBP2 expression (**Figure 1h**). This positive correlation was further supported by analysis of the DepMap database (**Figure 1i**). In addition, TMA analysis demonstrated that advanced-stage TNBC (Stage II-IV) exhibited a higher prevalence of eEF2K or PCBP2 overexpression compared with early-stage tumors (Stage I) (**Figure 1j and k**). Collectively, these results provide compelling clinically evidence for a positive correlation between eEF2K and PCBP2 in TNBC.

### eEF2K upregulates PCBP2 expression by suppressing its ubiquitin-proteasome-dependent degradation

**Figure 2:**
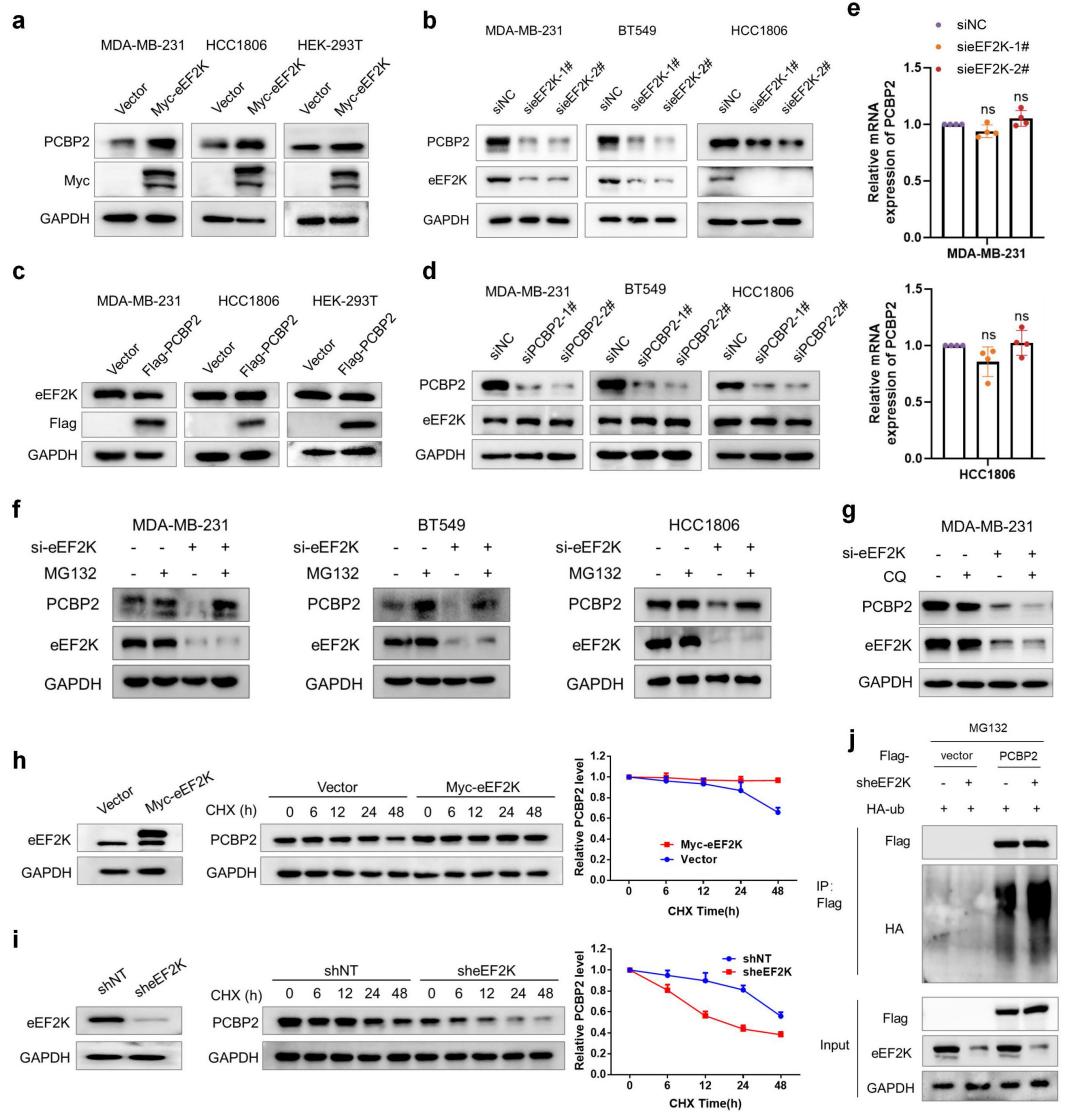
** eEF2K upregulates PCBP2 by inhibiting its ubiquitin-proteasomal degradation. a**) Western blot analysis of eEF2K and PCBP2 protein levels in MDA-MB-231, HCC1806 and HEK-293T cells transfected with Vector or Myc-eEF2K plasmids. **b**) Western blot analysis of eEF2K and PCBP2 protein levels 48 hours after transfection NC or eEF2K-targeted siRNAs in MDA-MB-231, HCC1806 and BT549 cells. **c**) Western blot analysis of eEF2K and Flag protein levels in MDA-MB-231, HCC1806 and HEK-293T cells transfected with Vector or Flag-PCBP2 plasmids. **d**) Western blot analysis of eEF2K and PCBP2 protein levels 48 hours after transfection NC or siRNA-PCBP2 in three TNBC cell lines. **e**) qRT-PCR analysis of the effects of eEF2K knockdown on PCBP2 mRNA expression in MDA-MB-231 and HCC1806 cells. ns, no significance. **f**) MDA-MB-231, HCC1806 and BT549 cells were transfected with NC or si-eEF2K and were treated with MG132 (20 μM) before extracting proteins. The PCBP2 and eEF2K protein expression levels were detected by Western blot. **g**) MDA-MB-231 cells were transfected with NC or si-eEF2K and were treated with CQ (20 μM) before extracting proteins. The PCBP2 and eEF2K protein expression levels were detected by Western blot. **h**) MDA-MB-231 cells with overexpressed eEF2K were treated with CHX (100 µg/ml) for a specified period of time, and the PCBP2 protein expression level was detected by Western blot. **i**) A stable MDA-MB-231 cell line with eEF2K knockdown was constructed by transfecting lentivirus. The above cells were treated with CHX (100 µg/ml) for a specified period of time, and the PCBP2 protein expression level was detected by Western blot. **j**) The HEK-293T cells were co-transfected with the indicated plasmids and treated with MG132 (20 μM) for 6 hours. Cell lysates were immunoprecipitated with anti-flag antibody and Western blot was used to detect the level of ubiquitination.

Given the significant positive correlation between eEF2K and PCBP2, we next sought to determine whether a reciprocal regulatory relationship exists between them. Ectopic eEF2K expression in MDA-MB-231, HCC1806, and HEK-293T cells resulted in a marked upregulation of PCBP2 protein levels (**Figure 2a**). Consistently, eEF2K silencing with two independent siRNAs reduced PCBP2 protein levels in TNBC cell lines (**Figure 2b**). On the contrary, PCBP2 overexpression or knockdown in these cell lines had no effect on eEF2K expression (**Figure 2c and d**). Notably, knockdown of eEF2K did not lead to obvious change in PCBP2 mRNA levels (**Figure 2e**), suggesting post-transcriptional regulation.

To elucidate the molecular mechanisms underlying eEF2K-mediated regulation of PCBP2, we examined whether eEF2K influences PCBP2 protein stability. Treatment with the proteasome inhibitor MG132 but not the lysosomal inhibitor chloroquine (CQ) rescued the downregulation of PCBP2 caused by eEF2K silencing in TNBC cell lines (**Figure 2f and g**). Moreover, cycloheximide (CHX)-based protein turnover assays demonstrated that overexpression of eEF2K significantly enhanced the protein stability of PCBP2, whereas eEF2K knockdown accelerated PCBP2 degradation (**Figure 2h and i**). Ubiquitination assays confirmed that eEF2K knockdown substantially enhanced polyubiquitin conjugation on PCBP2 (**Figure 2j**). Collectively, these findings suggest that eEF2K sustains PCBP2 protein abundance by preventing its ubiquitin-proteasome-mediated degradation.

### PCBP2 Ser189 phosphorylation promotes proliferation, migration and invasion of TNBC cells

**Figure 3:**
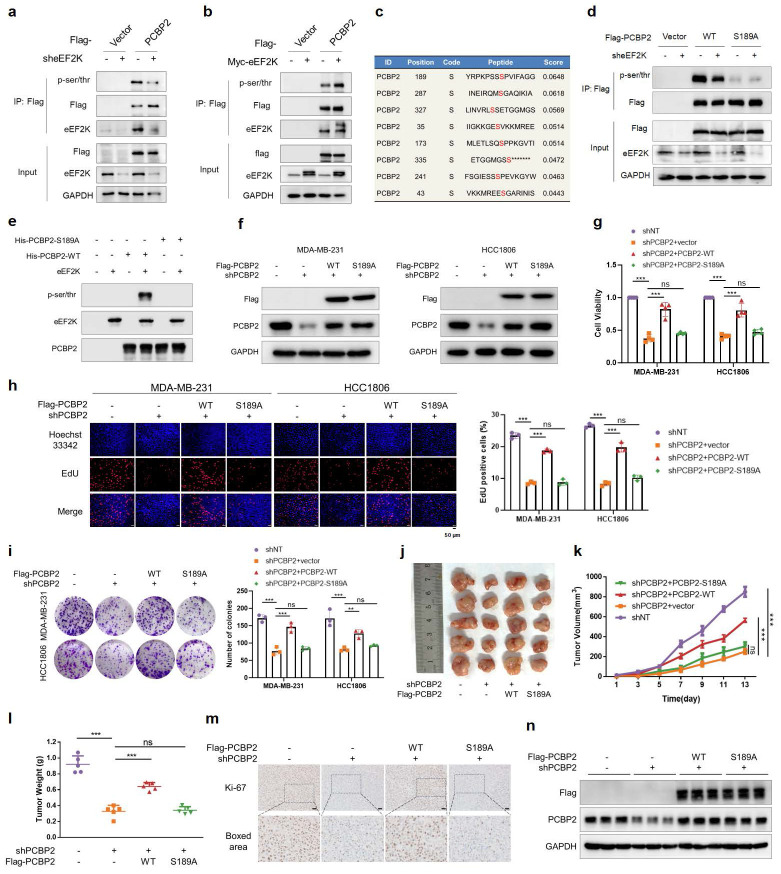
** Phosphorylation of PCBP2 Ser189 promotes proliferation of TNBC. a**) HEK293T cells were transfected with sh-eEF2K and Flag-PCBP2 plasmids, and then subjected to immunoprecipitation with anti-Flag antibody. The lysates and immunoprecipitates were then blotted. **b**) HEK293T cells were transfected with Myc-eEF2K and Flag-PCBP2 plasmids, and then subjected to immunoprecipitation with anti-Flag antibody. The lysates and immunoprecipitates were then blotted. **c**) The GPS 6.0 online prediction website (https://gps.biocuckoo.cn/index.php) was used to predict the specific sites and probabilities of potential phosphorylation of PCBP2 by eEF2K. **d**) HEK293T cells were transfected with sh-eEF2K, Flag-PCBP2-WT or Flag-PCBP2-S189A plasmids, and then subjected to immunoprecipitation with anti-Flag antibody. **e**) HEK293T cells were transfected with wildtype or S189A mutant of His-PCBP2. PCBP2 was immunoprecipitated by anti-His antibody, subsequently was dephosphorylated with lambda phosphatase and then incubated with or without a recombinant active eEF2K. The reaction mixtures were subjected to western blot with anti-pan-serine/threonine phosphorylation antibody.** f**) MDA-MB-231 and HCC1806 stable cell lines of shNT and shPCBP2 were established by lentivirus infection. Then, shPCBP2 cells were transfected with lentivirus to construct cells overexpressing Flag-PCBP2-WT and Ser189 locus mutant (S189A). The expression levels of PCBP2 and Flag in stable cell lines were detected by Western blot. **g**) After 48h of cell seeding, CCK8 assay was used to detect the proliferation rate of stable cell lines in (f). ns, no significance, ***p < 0.001. **h**) EdU assays were used to detect the proliferation rate of table cell lines in (f). ns, no significance, ***p < 0.001. Scale bar, 50 μm. **i**) The proliferative effects of the table cell lines in (f) were examined by colony formation assays. ns, no significance, **p < 0.01, ***p < 0.001. **j, k, l**) Stably expressed MDA-MB-231 cells in (f) were injected subcutaneously into nude mice. After one week, tumor volume was measured every other day, and the mice were executed two weeks later. Tumors were photographed and their weight measured. The values are presented as mean ± s. d. (n=5), ns, no significance, ***p < 0.001. **m**) Representative images of Ki67 staining of subcutaneous tumor sections. Scale bar, 50 μm. **n**) Western blot detects the expression levels of PCBP2 and Flag in subcutaneous graft tumors.

As an α-kinase, eEF2K exerts functional modulation through substrate phosphorylation. To investigate whether eEF2K phosphorylates PCBP2, we firstly conducted co-IP experiments in Flag-PCBP2-transfected HEK-293T cells and demonstrated that eEF2K downregulation could decrease the pan ser/thr phosphorylation level of PCBP2 protein (**Figure 3a**). Meanwhile, overexpression of eEF2K could conversely up-regulate the pan ser/thr phosphorylation level of PCBP2 protein (**Figure 3b**). Bioinformatic prediction using the GPS 6.0 revealed that Ser189 of PCBP2 is the most probable phosphorylation site by eEF2K compared to other residues (**Figure 3c**). Meanwhile, sequence analysis of PCBP2 identified Ser189 as an evolutionarily conserved phosphorylation site responsible for increased protein stability 31. It was observed that eEF2K has no effect on the phosphorylation level of PCBP2 with a Ser189 mutation (**Figure 3d**). An in vitro kinase assay further showed that activated eEF2K phosphorylates purified wild-type (WT) His-PCBP2, but not the S189A mutant (**Figure 3e**). Since PCBP2 plays an essential role in promoting the proliferation and migration in a variety of tumor cells 28, 29, 32-34, especially in human breast cancer cells 30. We therefore hypothesize that the oncogenic function of PCBP2 in TNBC is dependent on its phosphorylation at Ser189. To test this, we performed functional assays in MDA-MB-231 and HCC1806 cells. The results showed that knockdown of PCBP2 significantly inhibited cell viability, reduced the percentage of EdU-positive cells (indicative of S-phase cells), and impaired colony formation ability, all of which were rescued by reintroduction of wild-type PCBP2 (PCBP2-WT) but not by the phosphorylation-deficient mutant PCBP2-S189A (**Figure 3f-i**). Similarly, wound-healing and transwell assays demonstrated that PCBP2-WT, but not PCBP2-S189A, restored the migration and invasion defects caused by PCBP2 depletion (**Figure S1a-c**). Together, these findings suggest that phosphorylation at Ser189 is critical for PCBP2-driven proliferation and migration of TNBC cells.

To further validate these findings *in vivo*, we established a xenograft tumor model using MDA-MB-231 cells. PCBP2 knockdown markedly inhibited tumor growth, as reflected by reduced tumor volume, tumor weight, and Ki67 staining, whereas reconstitution with PCBP2-WT—but not PCBP2-S189A—restored tumorigenicity (**Figure 3j-m**). Additionally, we verified the intervention effect of PCBP2 expression in tumor tissues of each group (**Figure 3n**). These results demonstrate that phosphorylation of PCBP2 at Ser189 promotes TNBC proliferation both *in vitro* and *in vivo*.

### eEF2K drives TNBC progression through phosphorylating PCBP2 Ser189

**Figure 4:**
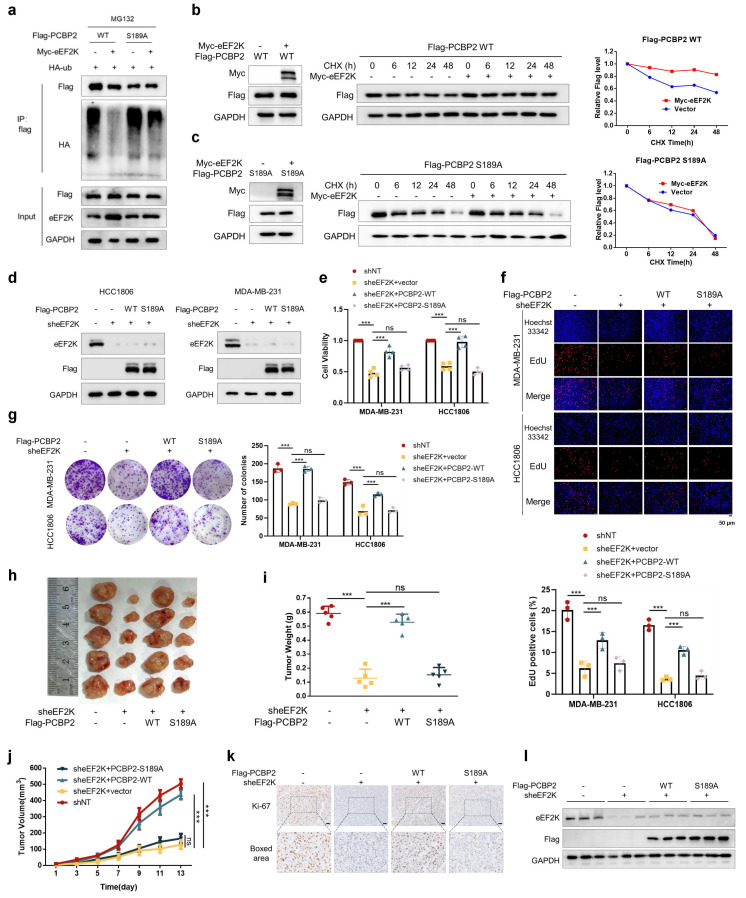
** eEF2K drives TNBC progression through phosphorylating PCBP2 Ser189. a**) HEK-293T cells were co-transfected with the indicated plasmids and treated with MG132 (20 μM) for 6 hours. Cell lysates were immunoprecipitated with anti-Flag antibody and Western blot was used to detect the level of ubiquitination. **b**) A stable cell line expressing Flag-PCBP2-WT was constructed in MDA-MB-231 cells stably overexpressing eEF2K. The above cells were treated with CHX (100 µg/ml) for a specified period of time, and the Flag protein expression level was detected by Western blot. **c**) A stable cell line expressing Flag-PCBP2-S189A was constructed in MDA-MB-231 cells stably overexpressing eEF2K. The above cells were treated with CHX (100 µg/ml) for a specified period of time, and the Flag protein expression level was detected by Western blot. **d**) Stable cell lines of MDA-MB-231 and HCC1806 were established by lentiviral infection. The expression levels of eEF2K and Flag in stable cell lines were detected by Western blot. **e**) After 48h of cell seeding, CCK8 assay was used to detect the proliferation rate of stable cell lines in (d). ns, no significance, ***p < 0.001. **f**) EdU assays were used to detect the proliferation rate of table cell lines in (d). ns, no significance, ***p < 0.001. Scale bar, 50 μm. **g**) The proliferative effects of the table cell lines in (d) were examined by colony formation assays. ns, no significance, ***p < 0.001. **h-j**) Stably expressed MDA-MB-231 cells in (d) were injected subcutaneously into nude mice. After one week, tumor volume was measured every other day, and the mice were executed two weeks later. Tumors were photographed and their weight measured. The values are presented as mean ± s. d. (n=5), ns, no significance, ***p < 0.001. **k**) Representative images of Ki67 staining of subcutaneous tumor sections. Scale bar, 50 μm. **l**) Western blot detects the expression levels of eEF2K and Flag in subcutaneous graft tumors.

Our results demonstrated eEF2K-mediated suppression of PCBP2 ubiquitination. To mechanistically link Ser189 phosphorylation with this stabilization, we found that ectopic eEF2K expression reduced polyubiquitination of PCBP2-WT but not the S189A mutant (**Figure 4a**). Consistently, CHX chase assays confirmed that overexpression of eEF2K significantly increased the protein stability of exogenously expressed Flag-PCBP2-WT, whereas the stability of Flag-PCBP2-S189A remained unaffected (**Figure 4b, c**). These results indicate that eEF2K stabilizes PCBP2 by phosphorylating it at Ser189.

Our research group has previously reported that knocking down eEF2K inhibits the proliferation and migration of TNBC cells 16. We intend to further investigate whether these oncogenic effects are mediated by PCBP2 phosphorylation. Rescue experiments in MDA-MB-231 and HCC1806 revealed PCBP2-WT, but not S189A, restored reduced proliferation rates, delayed S-phase progression, and weakened clonogenic capacity following eEF2K knockdown (**Figure 4d-g**). Functional complementation assays further demonstrated PCBP2-WT but not S189A specifically restored transwell migration and matrigel invasion capacities impaired by eEF2K knockdown (**Figure S2a-c**). These results suggest that eEF2K promotes TNBC cell proliferation and migration by phosphorylating PCBP2 at Ser189.

Consistently,* in vivo* xenograft models further recapitulated these *in vitro* findings: eEF2K silencing reduced tumor volume and tumor weight, reversible by PCBP2-WT but not S189A (**Figure 4h-j**). In addition, Ki67 staining of tumor sections showed that eEF2K knockdown significantly inhibited cell proliferation, and recombinant PCBP2-WT could restore cell proliferation, but recombinant PCBP2-S189A could not (**Figure 4k**). The protein expression of eEF2K and reconstructed Flag-PCBP2 in tumor tissues was consistent with the grouping (**Figure 4l**). In summary, our findings establish that eEF2K promotes the proliferation, migration, and invasion of breast cancer cells by phosphorylating PCBP2 at Ser189, highlighting the critical role of this regulatory axis in TNBC progression.

### eEF2K upregulates the mRNA levels of TNC, SOX5 and ITGB3 by phosphorylating PCBP2 Ser189

**Figure 5:**
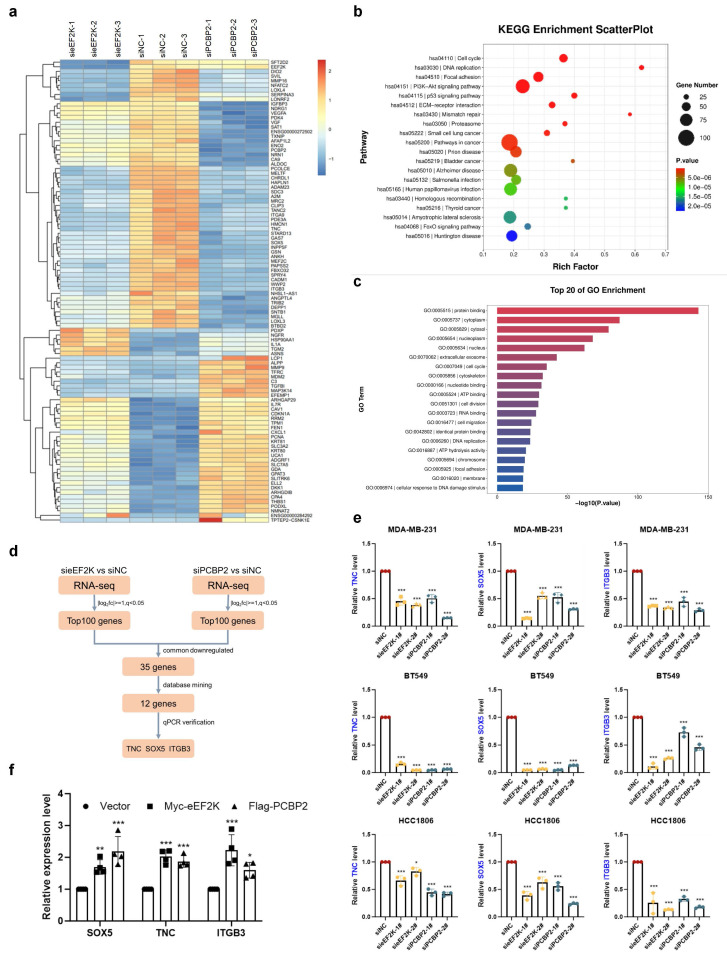
** RNA-seq reveals TNC, SOX5 and ITGB3 are downstream genes of eEF2K-PCBP2. a**) The RNA-seq heatmap of three pairs of siPCBP2, sieEF2K and siNC MDA-MB-231 cells. **b-c**) KEGG and GO Enrichment from RNA-seq. **d**) The Screening Process of Downstream Genes of eEF2K and PCBP2. **e**) qRT-PCR was performed to assess the mRNA expression of TNC, SOX5 and ITGB3 after transfection siNC, sieEF2K-1#, sieEF2K-1#, siPCBP2-1#, siPCBP2-2# target sequences in MDA-MB-231, HCC1806 and BT549. β-actin was the internal control. *p< 0.05, ***p< 0.001. **f**) qRT-PCR was conducted to evaluate the mRNA expression levels of TNC, SOX5 and ITGB3 following the overexpression of eEF2K and PCBP2 in MDA-MB-231 cells. β-actin was the internal control. *p< 0.05, **p< 0.01, ***p< 0.001.

To identify downstream signals of eEF2K-PCBP2, we performed RNA sequencing on MDA-MB-231 cells with eEF2K or PCBP2 knockdown. Using a significance threshold of q < 0.05, clustering heatmaps were generated for the significantly differentially expressed genes (**Figure 5a**). Gene ontology and KEGG pathway analyses revealed that eEF2K- and PCBP2-regulated genes were broadly enriched in cancer-associated pathways, including biological processes related to cell proliferation and migration (**Figure 5b-c**). Comparative analysis revealed 35 genes that were co-downregulated upon silencing of either eEF2K or PCBP2, among which 12 proliferation-related candidates were prioritized through literature review and database mining (**Figure 5d**). Subsequent qRT-PCR validation demonstrated that three of these genes—TNC, SOX5, and ITGB3—were consistently downregulated following eEF2K or PCBP2 depletion in MDA-MB-231, HCC1806, and BT549 cells (**Figure 5d-e and S3a-c**). Conversely, overexpression of PCBP2 or eEF2K in MDA-MB-231 cells led to upregulation of TNC, SOX5, and ITGB3 mRNA levels (**Figure 5f**).

**Figure 6:**
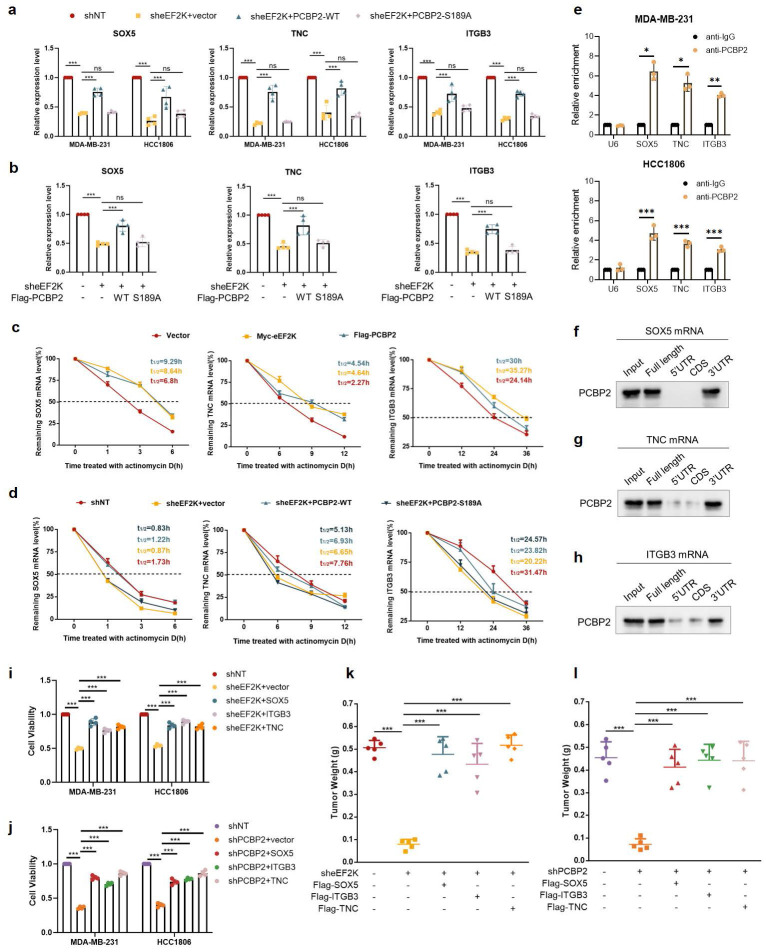
** eEF2K maintains mRNA stability of TNC, SOX5 and ITGB3 by phosphorylating PCBP2 Ser189. a**) qRT-PCR was conducted to evaluate the mRNA expression of TNC, SOX5 and ITGB3 in MDA-MB-231 and HCC1806 stable cell lines transfected with either PCBP2-WT or PCBP2-S189A lentivirus following eEF2K knockdown. β-actin served as the internal reference gene. ns, no significance, ***p< 0.001. **b**) The mRNA expression levels of SOX5, TNC and ITGB3 were quantified by qRT-PCR in the tumor tissues derived from the mouse model depicted in Figure 4h. β-actin was the internal control. ns, no significance, ***p< 0.001. **c**) 2.5 μg/mL ActD were used to block mRNA transcription and qRT-PCR was used to detect the effects of eEF2K and PCBP2 overexpression on the degradation rate of TNC, SOX5 and ITGB3 mRNA in MDA-MB-231 cells. 18S was the internal control. **d**) MDA-MB-231 cells were treated with 2.5 μg/mL ActD to block mRNA transcription. qRT-PCR was performed to determine the effects of PCBP2-WT and PCBP2-S189A lentivirus transfection, following eEF2K knockdown, on the degradation rates of TNC, SOX5 and ITGB3 mRNA. 18S was the internal control. **e**) In MDA-MB-231 and HCC1806 cells, RIP was performed using anti-PCBP2 and control IgG antibodies, followed by qRT-PCR to examine the enrichment of SOX5, TNC, ITGB3 and U6. U6 served as negative control, *p < 0.05, **p < 0.01, ***p < 0.001. **f-h**) RNA pull-down assay was employed to identify the binding fragments of SOX5 mRNA, TNC mRNA or ITGB3 mRNA with PCBP2. **i-j**) sheEF2K or shPCBP2 MDA-MB-231 and HCC1806 cells were transfected with lentivirus to construct cells overexpressing Flag-TNC, Flag-SOX5, and Flag-ITGB3. After 48h of cell seeding, CCK8 assay was used to detect the proliferation rate of stable cell lines. ***p < 0.001. **k**) Stably expressed MDA-MB-231 cells in (i) were injected subcutaneously into nude mice. The weight of the subcutaneous tumors in the mice was measured in the end. The values are presented as mean ± s. d. (n=5), ***p < 0.001. **l**) Stably expressed MDA-MB-231 cells in (j) were injected subcutaneously into nude mice. The weight of the subcutaneous tumors in the mice was measured in the end. The values are presented as mean ± s. d. (n=5), ***p < 0.001.

Building on our earlier findings that eEF2K phosphorylates PCBP2 at Ser189 to enhance its protein stability, we next investigated whether this regulatory axis influences the mRNA levels of TNC, SOX5, and ITGB3. QRT-PCR analysis demonstrated that the reduction of TNC, SOX5, and ITGB3 transcripts caused by eEF2K knockdown was restored by PCBP2-WT but not by the S189A mutant (**Figure 6a**). A similar trend was observed in subcutaneous tumor tissues from the xenograft model described in Figure 4h, where PCBP2-WT, but not S189A, rescued the downregulation of these mRNAs following eEF2K depletion (**Figure 6b**).

As an RNA-binding protein, PCBP2 binds to the 3'UTR of target mRNAs to stabilize transcripts of downstream genes 26, 35. To explore the effect of eEF2K-PCBP2 axis on mRNA stability, actinomycin D chase assays revealed that overexpression of eEF2K or PCBP2 significantly extended the half-lives of TNC, SOX5, and ITGB3 mRNAs in MDA-MB-231 cells (**Figure 6c**). Conversely, eEF2K knockdown accelerated their decay, which was rescued by PCBP2-WT but not PCBP2-S189A (**Figure 6d**). RIP assays further confirmed the direct interaction between PCBP2 and the mRNAs of TNC, SOX5, or ITGB3, showing robust enrichment upon anti-PCBP2 immunoprecipitation (**Figure 6e**). In addition, RNA pull-down assays demonstrated that PCBP2 selectively bound to biotin-labeled full-length or 3'UTR fragments, but not to the 5'UTR or coding sequences (CDS), of SOX5 mRNA (**Figure 6f**). Similar binding patterns were observed for TNC and ITGB3, where PCBP2 interacted with their full-length transcripts and 3'UTRs (**Figure 6g-h**). Rescue experiments further demonstrated that knockdown of eEF2K or PCBP2 inhibited TNBC proliferation both *in vitro* and* in vivo*, whereas this inhibitory effect was reversed by the overexpression of TNC, SOX5, or ITGB3, respectively (**Figure 6i-l and Figure S4a-d**) indicating that TNC, SOX5 and ITGB3 function as essential downstream effectors of eEF2K/PCBP2 axis. Together, these findings establish that eEF2K orchestrates an oncogenic transcriptome by phosphorylating PCBP2 at Ser189 to stabilize malignancy-associated mRNAs, revealing a post-transcriptional mechanism driving TNBC progression.

### Coordinated eEF2K-PCBP2 expression correlates with oncogenic transcripts in TNBC

**Figure 7:**
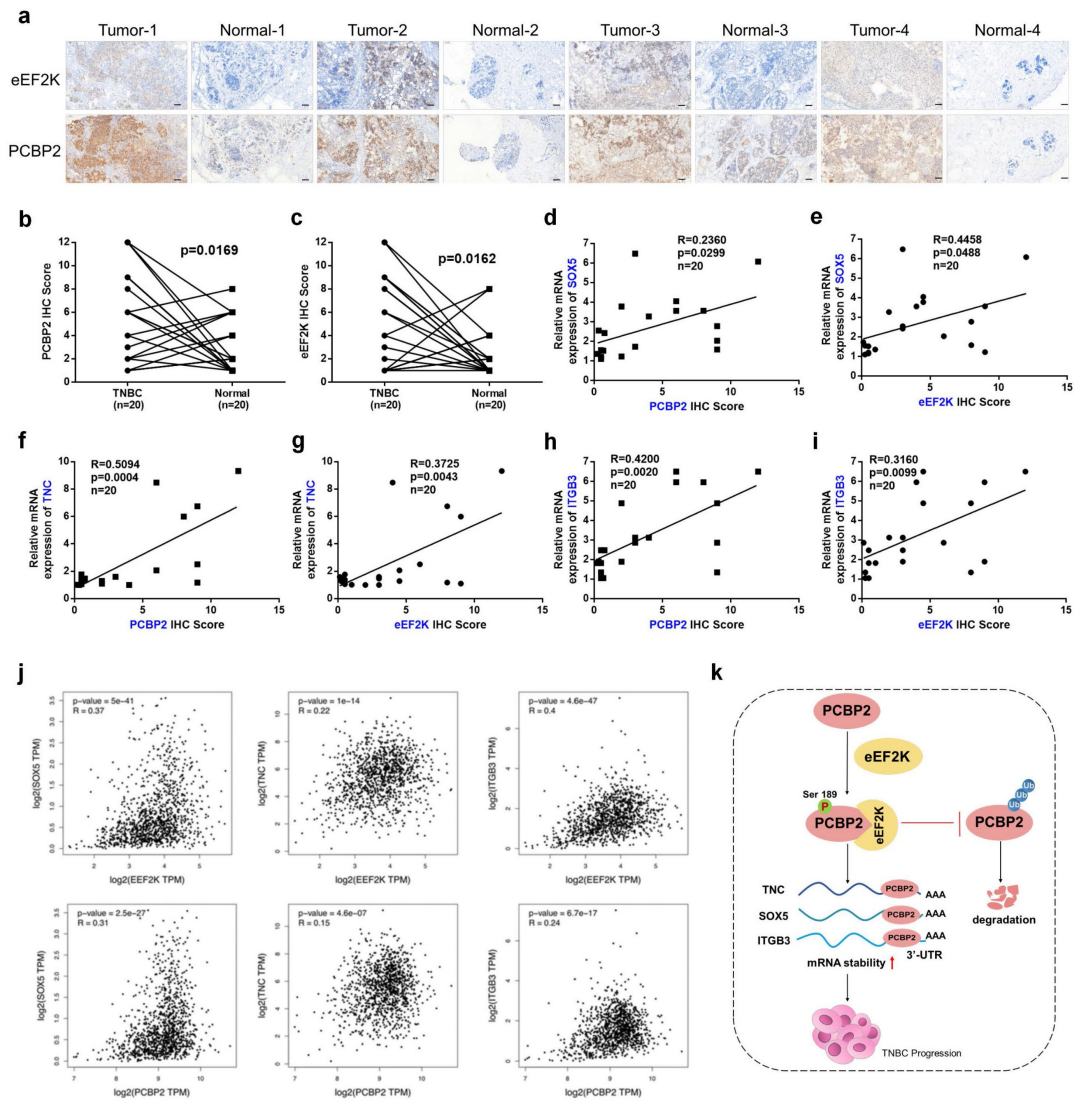
** Coordinated eEF2K-PCBP2 expression correlates with oncogenic transcripts in TNBC. a-c**) 20 pairs of TNBC and adjacent tissues were detected by immunohistochemistry using anti-eEF2K and anti-PCBP2 antibodies. Semi quantitative scoring method (using a scale from 0 to 12) was used to analyzed the scores of eEF2K and PCBP2 IHC staining. The relative IHC scores of eEF2K and PCBP2 were calculated using the IHC scores of each pair of adjacent tissues as the standard. Data analysis was performed using Paired sample t-test and Pearson's correlation analysis. Representative images of eEF2K and PCBP2 in TNBC and adjacent tissues. Scale bar, 100 μm. **d-i**) The mRNA expressions of SOX5, TNC and ITGB3 in 20 pairs of TNBC and adjacent tissues were detected by qRT-PCR. Correlation of RNA expression levels of SOX5, TNC and ITGB3 with IHC scores of eEF2K and PCBP2, respectively. **j**) Analyze the correlations between eEF2K/PCBP2 and TNC/SOX5/ITGB3 mRNA expression in breast cancer tissues and adjacent normal tissues using the GEPIA database. **k**) The schematic diagram illustrates the mechanism by which eEF2K increases PCBP2 in TNBC. eEF2K promotes the phosphorylation of the PCBP2 Ser189 site, stabilizing TNC, SOX5 and ITGB3 mRNA, thereby facilitating the progression of TNBC cells.

To evaluate the clinical relevance of eEF2K and PCBP2 in breast cancer tissues, IHC staining was conducted on paired samples of TNBC tissues and matched adjacent non-tumor tissues. Consistent with our earlier observations, quantitative scoring revealed elevated eEF2K and PCBP2 protein abundance in tumors versus normal tissues (**Figure 7a-c**). In the same cohort, qRT-PCR profiling demonstrated positive correlations between eEF2K IHC scores and TNC/SOX5/ITGB3 transcript levels, as well as between PCBP2 protein abundance and the expression of these three genes (**Figure 7d-i**). Analysis via the GEPIA database further revealed positive correlations between eEF2K/PCBP2 and the mRNA expression of downstream genes TNC, SOX5, and ITGB3 in breast cancer tissues relative to adjacent normal (**Figure 7j**). The aforementioned findings demonstrate a significant positive correlation between the expression levels of eEF2K, PCBP2 and the mRNA levels of TNC, SOX5, and ITGB3 in clinical samples. These concordance between protein correlation signatures and transcriptional outputs in patient specimens underscores the clinical relevance of the eEF2K-PCBP2-TNC/SOX5/ITGB3 axis, reinforcing their mechanistic role in TNBC progression.

## Discussion

Due to the lack of specific targets and rapid clinical progression, the treatment of TNBC is still dominated by chemotherapy and inevitable resistance development, necessitating urgent exploration of mechanism-driven targeted strategies 36. In a variety of malignant tumors, the overexpression of eEF2K promotes the proliferation, migration and invasion of cancer cells 20, 37. In this study, we found that eEF2K-mediated phosphorylation of PCBP2 at Ser189 inhibits its ubiquitination-dependent degradation, thereby stabilizing TNC, SOX5, and ITGB3 mRNAs and ultimately promoting TNBC progression (**Figure 7k**).

Extensive research has focused on the upstream signaling pathways of eEF2K. For example, mTORC1 and ERK cooperatively phosphorylate eEF2K at Ser358 to inhibit its activity 17, while protein kinase A, activated by cyclic adenosine monophosphate, phosphorylates eEF2K at Ser500 to suppress protein synthesis 38. In contrast, studies on the downstream substrates of eEF2K are limited. In this study, we identified PCBP2 as a potential eEF2K-interacting protein through IP-MS screening, and confirmed their specific binding in TNBC cells via Co-IP assays. Analyses of TNBC patient samples and the DepMap database further demonstrated a significant positive correlation between eEF2K and PCBP2 expression. We further found that silencing or overexpressing eEF2K in TNBC cells altered PCBP2 protein abundance without affecting its mRNA levels, indicating that eEF2K regulates PCBP2 through a post-transcriptional mechanism. Importantly, this regulation operates unidirectionally, as PCBP2 modulation failed to impact eEF2K expression, establishing eEF2K as the upstream regulator in this pathway.

PCBP2 plays a carcinogenic role in a variety of cancers, such as glioblastoma, colorectal cancer, and gastric cancer 28, 39, 40. Wu et al. found that PCBP2 overexpression was associated with disease progression and poor prognosis in breast cancer 30. Liu et al. found that PCBP2 stabilized squalene epoxidase mRNA, activated PI3K/Akt signaling, and controlled cell stemness in breast cancer 41. However, how to control the mechanism of action of PCBP2 in breast cancer has not been reported. Our study identifies eEF2K as a novel upstream regulator of PCBP2 in TNBC. Previous work by Chang et al. demonstrated that ERK1/2 signaling stabilizes PCBP2 through multisite phosphorylation at conserved serine and threonine residues, including Ser189 31. Consistent with this, our bioinformatic analysis predicted Ser189 as a high-confidence phosphorylation site and a likely site specifically targeted by eEF2K for phosphorylation. we previous found that eEF2K phosphorylates PCBP2 at Ser189, thereby regulating the protein stability of PCBP2. This finding is consistent with our previous studies demonstrating that eEF2K phosphorylates GSK3β at Ser9 20 and AURKA at Ser391 21 to modulate their protein stability. We further provide the first experimental evidence that eEF2K phosphorylates PCBP2 at Ser189, thereby preventing its ubiquitin-proteasome-mediated degradation. This finding expands the substrate spectrum of eEF2K, which was previously thought to act almost exclusively on eEF2.

Overexpression of PCBP2 significantly enhances the proliferation, migration and invasion of TNBC cells 30. The lncRNA ST8SIA6-AS1 has been shown to facilitate colorectal cancer progression through PCBP2-mediated mechanisms, driving increased tumor cell proliferation and metastatic potential including enhanced migration and invasion 34. In this study, we revealed that phosphorylation at the Ser189 site of PCBP2 promotes these malignant behaviors in TNBC, as evidenced by both cellular and animal experiments. Our research group previously demonstrated that eEF2K promotes TNBC progression by phosphorylating AURKA at Ser391 21. This study demonstrates through comprehensive in vivo and in vitro experiments that eEF2K promotes TNBC progression by phosphorylating PCBP2 at Ser189, thereby enhancing tumor cell proliferation, migration and invasion.

Building on established roles of PCBP2 in mRNA stability regulation 27, RNA-seq analysis identified genes that were co-downregulated following the knockdown of eEF2K and PCBP2, and these findings were validated in three TNBC cell lines. This approach revealed three clinically relevant targets - TNC, SOX5 and ITGB3 - all previously associated with breast cancer proliferation 42-44. While eEF2K has been implicated in regulating tumor cell survival, metabolism, and motility, its involvement in post-transcriptional gene regulation through RNA-binding proteins has not been reported. Our studies demonstrated that knockdown of eEF2K reduced the mRNA levels of TNC, SOX5, and ITGB3, which were restored by reconstitution with wild-type PCBP2 but not the Ser189A mutant. Collectively, these results demonstrate that the eEF2K-PCBP2 axis orchestrates the stability of malignancy-associated mRNAs, providing a mechanistic explanation for TNBC progression at the post-transcriptional level. The oncogenic significance of TNC, SOX5, and ITGB3 is well established: TNC drives EMT-mediated progression 42, ITGB3 enhances viability/motility while suppressing apoptosis 45, and SOX5 promotes TNBC via the circ_0084653-USP36 axis 44. Clinically, we observed significant positive correlations between eEF2K/PCBP2 expression and TNC, SOX5, and ITGB3 mRNA levels in TNBC patient samples, which were supported by independent database analyses. Consistent with previous reports of PCBP2 binding to 3' UTRs to stabilize mRNAs, such as NCAPG2 46, UFD1, and NT5E 30, we show that PCBP2 specifically binds to the 3' UTRs of TNC, SOX5, and ITGB3, thereby modulating their transcript stability. Together, these findings establish the eEF2-PCBP2 axis as a key post-transcriptional regulator of oncogenic mRNAs in TNBC, with direct validation in clinical samples.

In summary, this study reveals uncovers a previously unrecognized oncogenic mechanism whereby eEF2K phosphorylates PCBP2 at Ser189, blocking its ubiquitin-proteasome degradation and enhancing protein stability. This post-translational regulation subsequently stabilizes TNC, SOX5, and ITGB3 mRNAs, ultimately driving TNBC cell proliferation, migration and invasion. Our results identify a novel eEF2K-mediated pathway driving TNBC progression, establishing a rationale for developing eEF2K-targeted therapies.

## Supplementary Material

Supplementary figures and table.
