# Supplementary material for: eEF2K-Mediated Stabilization of PCBP2 Promotes Oncogenic mRNA Programs in Triple-Negative Breast Cancer
Source: Int J Biol Sci. 2026 Mar 25;22(7):3714–30. doi: 10.7150/ijbs.127111 (PMC13086004; doi:10.7150/ijbs.127111)
Supplement: Supplementary file 1 — Supplementary figures and table. [file ijbsv22p3714s1.pdf]

Supporting information

eEF2K-Mediated Stabilization of PCBP2 Promotes Oncogenic mRNA Programs in Triple-Negative Breast Cancer

Yueying Cheng *[1,2], Ting Jiang *[1,2], Wenqian Zhu [3], Linhao He [1,2], Zongling Chen [4], Hang Li [1,2], Ruigang Zhao [5], Shilong Jiang ‡[6,7], Yan Cheng ‡[1,2,8,9,10,11]

[1] Department of Pharmacy, The Second Xiangya Hospital, Central South University, Changsha, 410011, China.

[2] Hunan Provincial Engineering Research Centre of Translational Medicine and Innovative Drug, Changsha 410011, China.

[3] Department of Medical Administration, The Second Hospital of Shandong University, Jinan, 250033, China.

[4] Department of General Surgery, The Second Xiangya Hospital, Central South University, Changsha, Hunan, 410011, China.

[5] Information and Network Center, Central South University, Changsha 410083, China.

[6] Department of Pharmacy, Xiangya Hospital, Central South University, Changsha, 410008, China.

[7] The Hunan Institute of Pharmacy Practice and Clinical Research, Xiangya Hospital, Central South University, Changsha, Hunan, 410008, China.

[8] Clinical Research Center For Breast Disease In Hunan Province, Changsha, 410011, China.

[9] FuRong Laboratory, Changsha 410078, Hunan, China.

[10] NHC Key Laboratory of Cancer Proteomics & State Local Joint Engineering Laboratory for Anticancer Drugs, Xiangya Hospital, Central South University, Changsha 410008, Hunan, China.

[11] Key Laboratory of Diabetes Immunology (Central South University), Ministry of Education Changsha, 410011, China.

*These authors contributed equally to this work.

‡ Address correspondence to: Yan Cheng, Department of Pharmacy, The Second Xiangya Hospital, Central South University, Changsha, China, E-mail: yancheng@csu.edu.cn. Shilong Jiang, Department of Pharmacy, Xiangya Hospital, Central South University, Changsha, China, E-mail: shilong@csu.edu.cn.

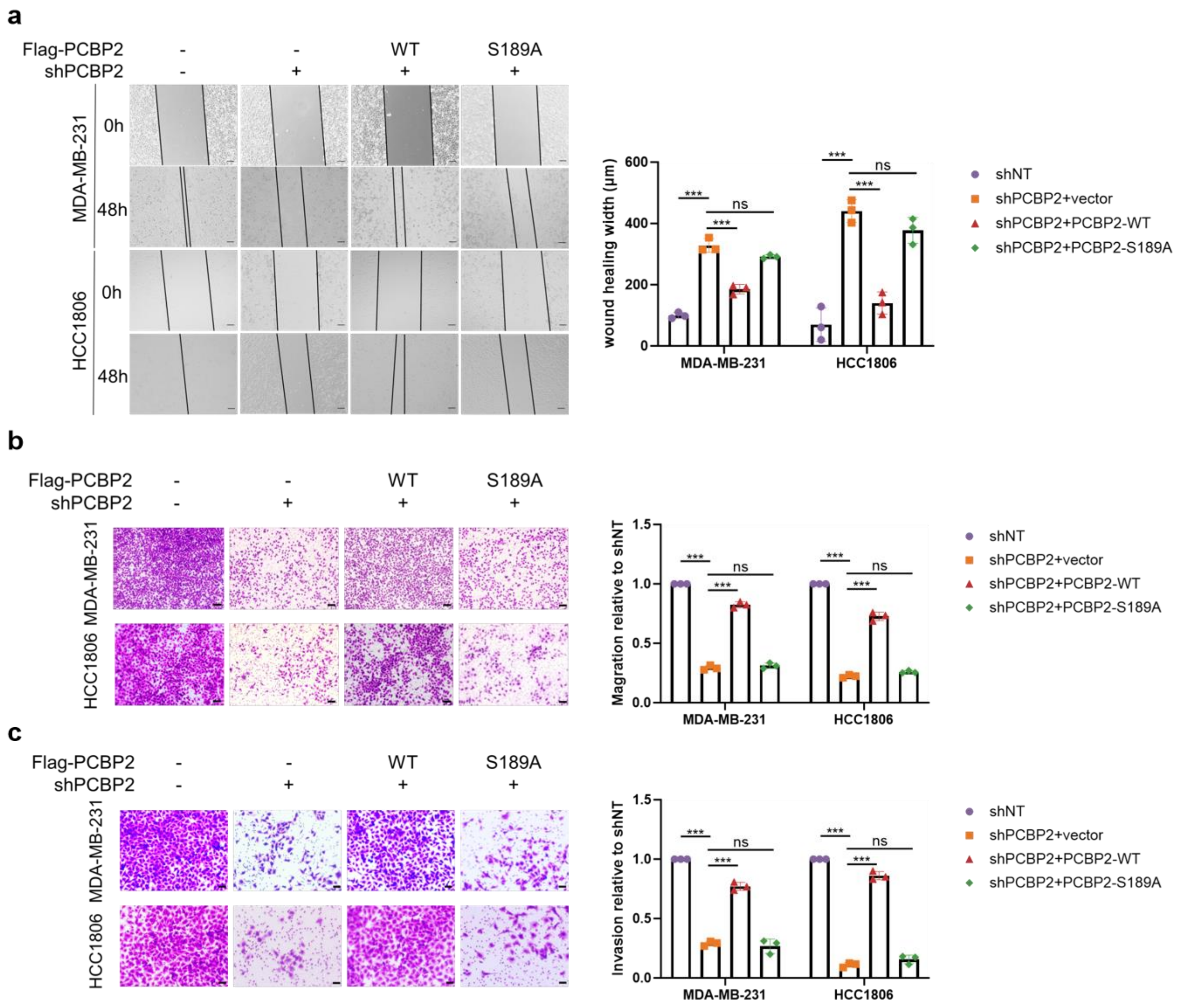

Figure S1. Phosphorylation of PCBP2 Ser189 promotes migration and invasion of TNBC cells. a) Scratch assay was done to show the effect stable cell lines in Figure 3f on cell migration ability. Scale bar, 100 μm. ns, no significance, ***p < 0.001. **b**) Representative images and the migration capacity of the stable cell lines shown in Figure 3f were determined through transwell chambers. Scale bar, 100 μm. ns, no significance, ***p < 0.001. **c**) Representative images and invasiveness measurements were performed on the stable cell lines shown in Figure 3f. Scale bar, 50 μm. ns, no significance, ***p< 0.001.

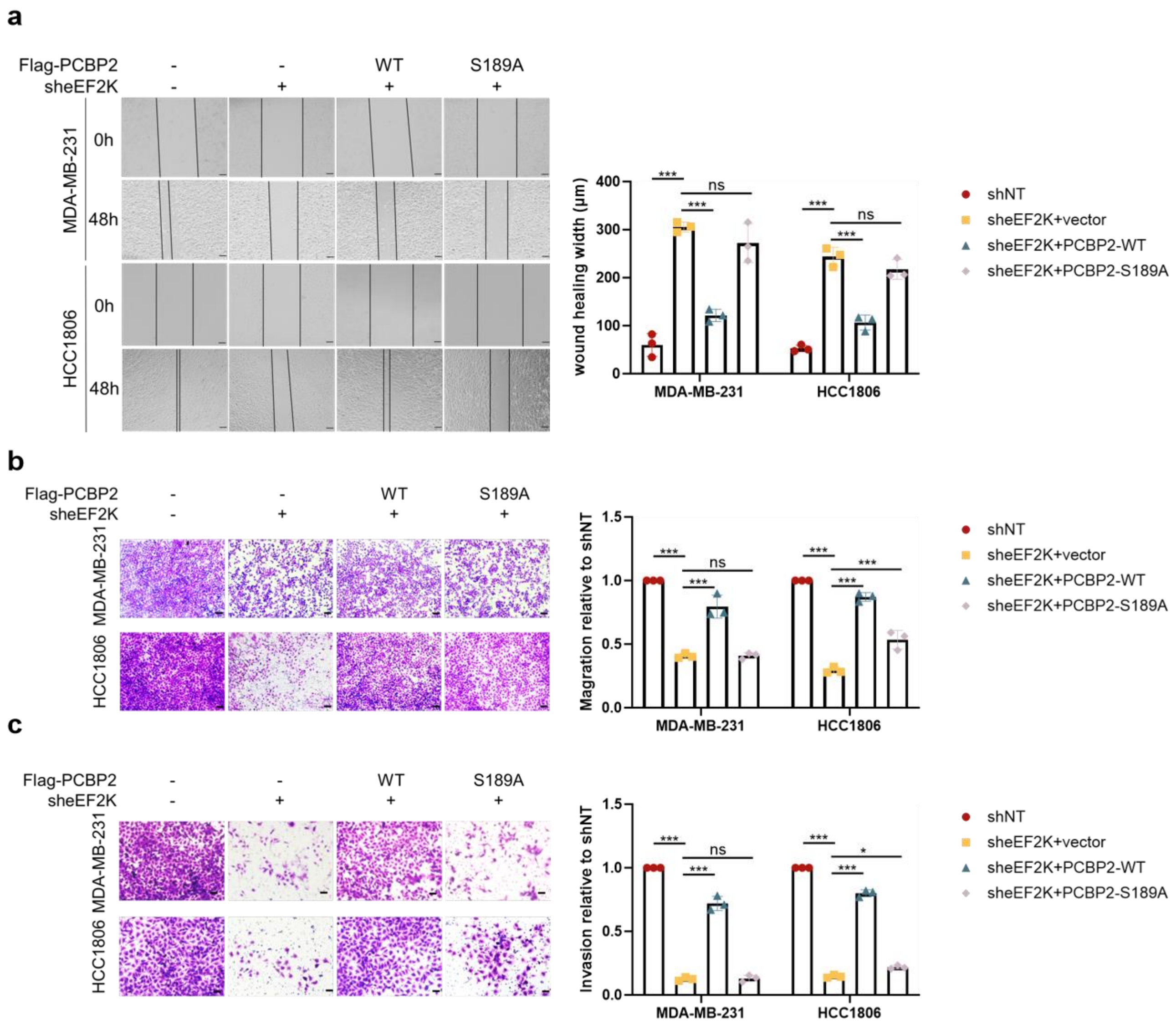

Figure S2. eEF2K promotes migration and invasion of TNBC cells by phosphorylating PCBP2 Ser189. **a**) Scratch assay was done to show the effect stable cell lines in Figure 4d on cell migration ability. Scale bar, 100 μm. ns, no significance, ***$p < 0.001$. **b**) Representative images and the migration capacity of the stable cell lines shown in Figure 4d were determined through transwell chambers. Scale bar, 100 μm. ns, no significance, ***$p < 0.001$. **c**) Representative images and invasiveness measurements were performed on the stable cell lines shown in Figure 4d. Scale bar, 50 μm. ns, no significance, *$p < 0.05$, ***$p < 0.001$.

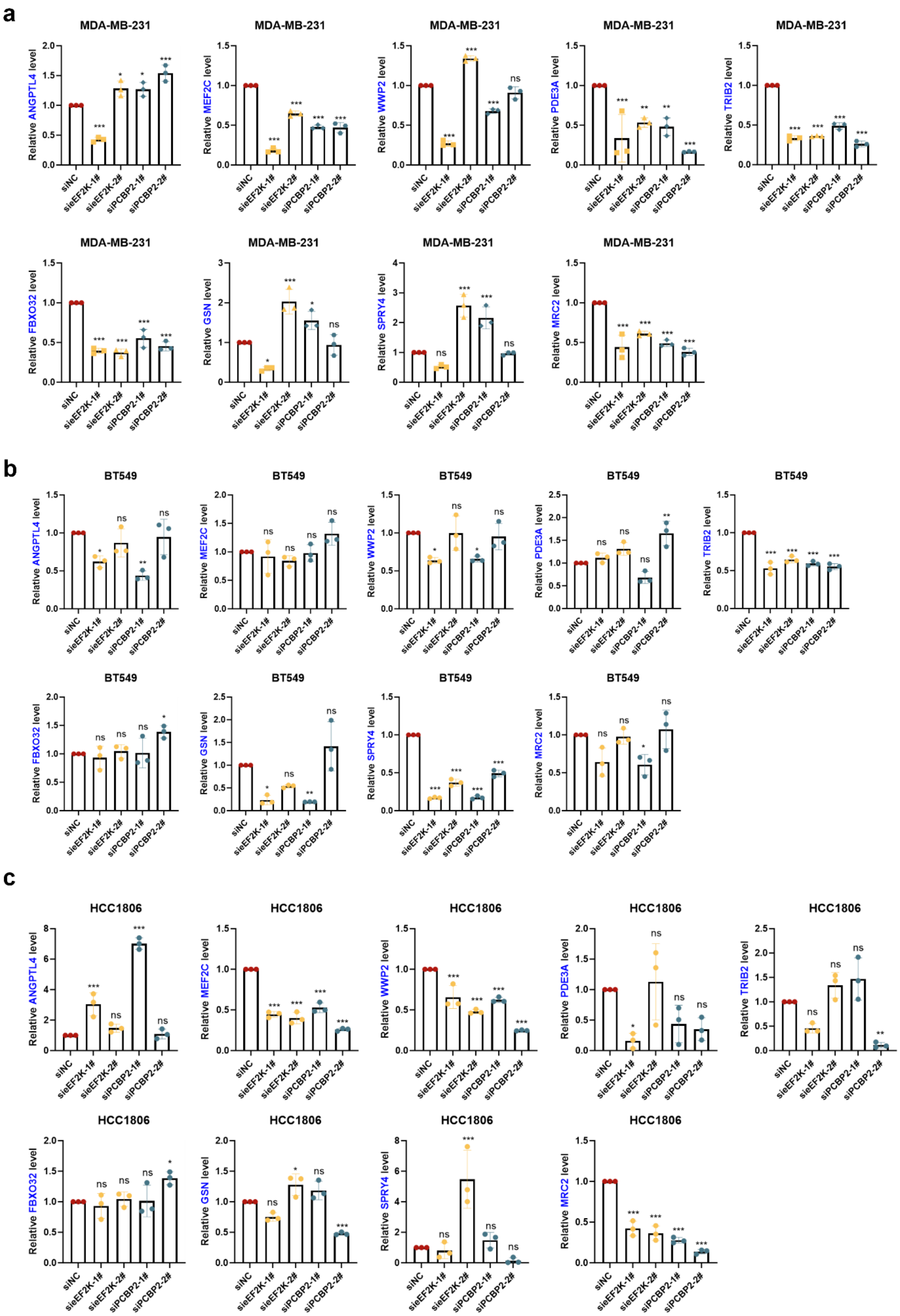

Figure S3. Verification of downstream genes of eEF2K and PCBP2. a-c) qRT-PCR was performed to assess the mRNA expression of nine genes after transfection NC, sieEF2K-1#, sieEF2K-1#, siPCBP2-1#, siPCBP2-2# target sequences in MDA-MB-231 (**a**), BT549 (**b**), and HCC1806 (**c**). β-actin served as the internal reference gene. ns, no significance, *$p < 0.05$, **$p < 0.01$, ***$p < 0.001$.

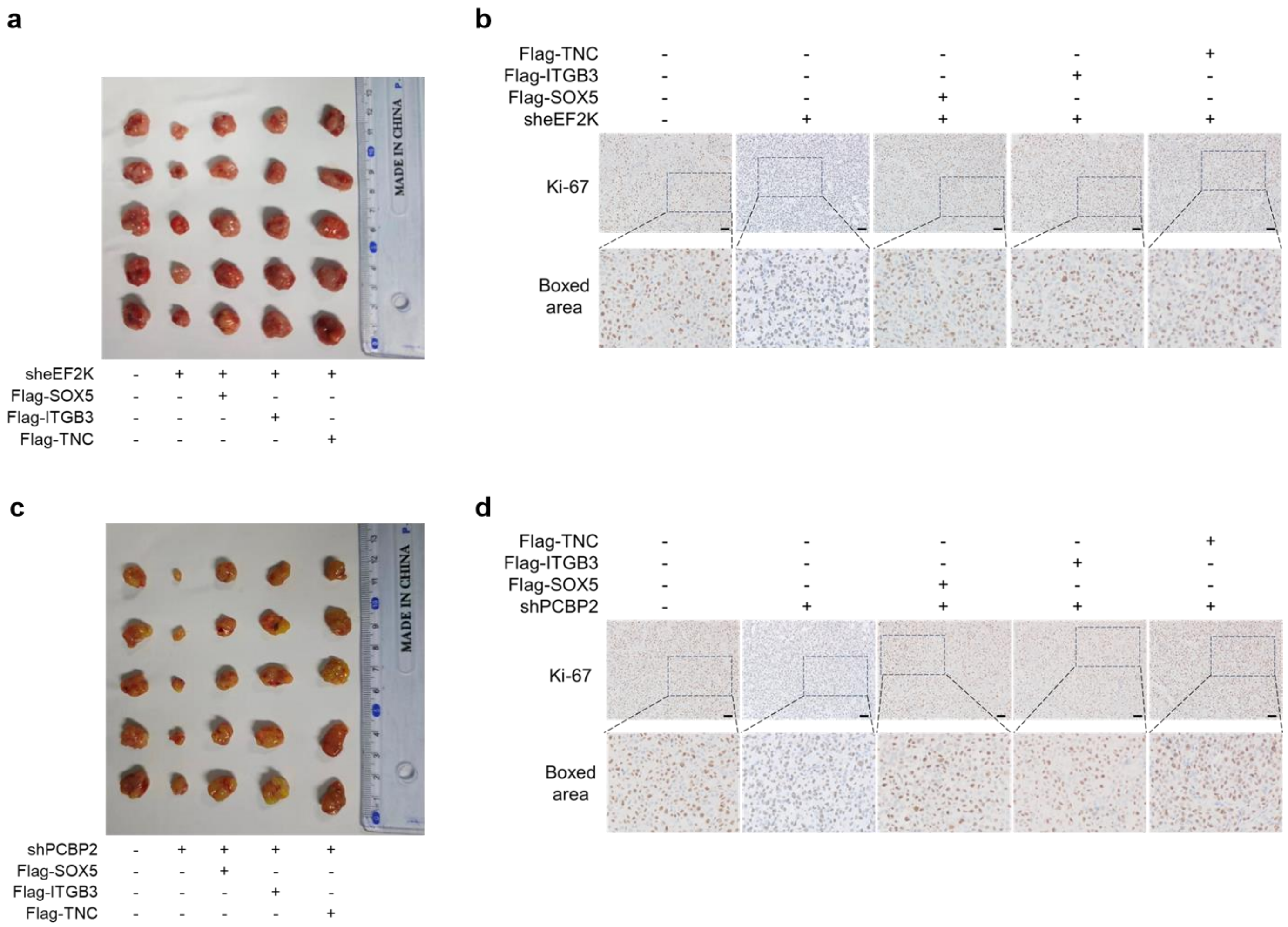

Figure S4. eEF2K and PCBP2 promote proliferation of TNBC through TNC, ITGB3 and SOX5. a) Tumors in Figure 6k were photographed. **b**) Representative images of Ki67 staining of subcutaneous tumor sections in (a). Scale bar, 50 μm. **c**) Tumors in Figure 6l were photographed. **d**) Representative images of Ki67 staining of subcutaneous tumor sections in (c). Scale bar, 50 μm.

Table S1. Target sequences of shRNAs and siRNAs.

siRNA target sequence	
siNC	Tsingke
eEF2K siRNA 1#	GCUCGAACCAGAAUGUCAA
eEF2K siRNA 2#	GCAAACUCCUUCCACUUCA
shRNA target sequence	
shNT	TTCTCCGAACGTGTCACGT
PCBP2 shRNA 1#	GCCATCACTATTGCTGGCATT
PCBP2 shRNA 2#	CCATGATCCATCTGTGTAGTT
